# Transforming custodial healthcare: A reflective summary of insights from my placement in Australia

**DOI:** 10.51866/mol.995

**Published:** 2025-11-17

**Authors:** Suriya Devi Gurunathan

**Affiliations:** 1 MD, FRACGP, Klinik Kesihatan Indera Mahkota, Jalan IM4, Bandar Indera MAhkota, Kuantan, Pahang Malaysia. Email: suriyadevi1171982@yahoo.com

**Keywords:** Prisoners/health services, Delivery of healthcare, Interprofessional relations, Continuity of patient care

Detainees are among the most medically and socially vulnerable populations, often presenting with a complex mix of untreated chronic diseases, mental health conditions, substance use disorders and infectious diseases. These issues are compounded by systemic stigma and historically limited access to care. As a general practitioner managing both community and custodial health services in Indera Mahkota, Pahang, I embarked on a 3-month placement with the Justice Health and Forensic Mental Health Network (JHFMHN) in New South Wales, Australia, alongside academic work at Western Sydney University, to explore effective custodial healthcare mode.

**Figure 1:**
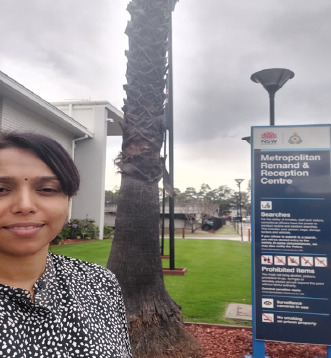
My visit to the Metropolitan Reception and Remand Centre in Silverwater, New South Wales. This facility served as a key site for entry health assessments and coordination of care across custodial settings.

**Figure 2:**
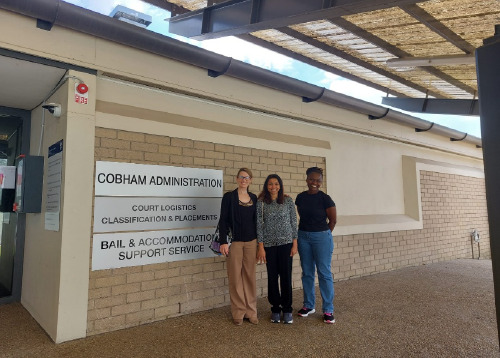
Visit to the Youth Justice Centre. Pictured on the right is Professor Le.gh Haysom, Clinical Director of Adolescent Health and Senior Staff Specialist Paediatrician, who provided insights into the delivery of adolescent health services within custodial settings.

From November 2024 to February 2025, I was immersed in a structured attachment that enabled me to observe clinical care delivery across a range of correctional environments, such as adult male and female prisons including the reception and remand centre (Figure 1), Youth Justice Centres (Figure 2), Long Bay Hospital and the Forensic Hospital. I witnessed the vital role of entry health assessments in identifying urgent medical needs and the delivery of 24/7 acute and chronic care through a well-coordinated network of onsite clinics, satellite services and remote specialist on-call support. Nurses, allied health professionals and specialists across the four core domains - primary care, mental health, addiction medicine and infectious diseases (population health) - played a central role in providing timely, person-centred care. I also observed how hospital-based specialist outreach services and virtual care effectively addressed barriers to care, such as legal, logistical and security constraints, by delivering essential services directly -within custodial settings.

Preventive care was thoughtfully embedded across every stage of the detainee healthcare journey - from initial intake to release. This included routine screening for bloodborne viruses and sexually transmitted infections, chronic disease management, vaccination programmes and harm reduction strategies. Young people in custody often present with significantly poorer physical and mental health, and Family Medicine Specialist Association of Malaysia alongside high rates of trauma,abuse, neglect and substance use. Many also face challenges such as low literacy, limited cognitive abilities and ponr educational attainment. Hie custodial setting plays a crucial role in recognising and addressing these issues early, offering a window of opportunity for intervention with the broader goal of reducing recidivism. Similarly, I came to appreciate the JHFMHN’s recognition of the unique, gender-specific healthcare needs of women in custody - many of whom have experienced a history of victimisation - through the provision of trauma-informed, culturally responsive card.

**Figure 3:**
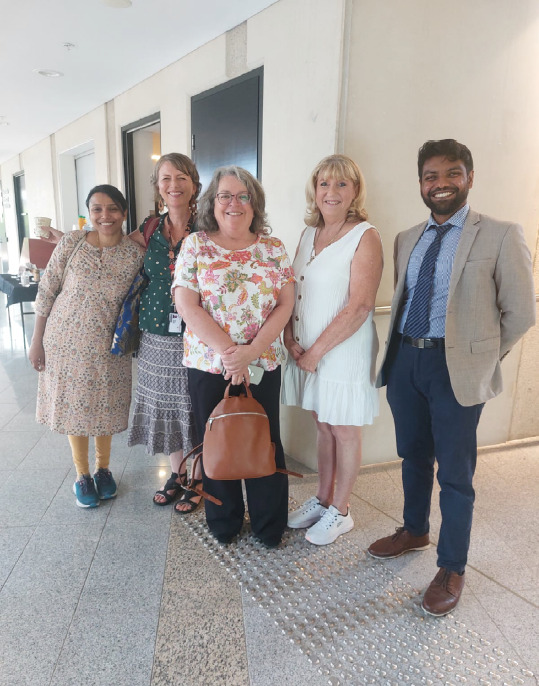
A moment from my academic engagement at Western Sydney University. The sacond person from the left is Professor Penelope Abbott, Peter Brennan Chair of General Practice, based at the School of Medicine, Campbelltown Campus, Western Sydney University, who provided academic supervision and mentorship throughout the placement.

My academic attachment at Western Sydney University further enriched my experience. As a ‘visiting fellow, I engaged in teaching and peer discussionsand developed a programme logic model to identify strategic priorities for reform. He integration of clinical exposure with academic reflection allowed me to appreciate how structured, preventive and collaborative care models in custodial settings can bridge health inequities and promote continuity of care beyond prison walls (Figure 3).

This experience has strengthened my resolve to advocate for improved custodial healthcare in my own context, with a focus on system-based approaches that prioritise early intervention, multidisciplinary collaboration, easy access and compassionate, equitable care.

